# Therapeutic potential of AdipoRon in cognitive, depressive, and anxiety disorders: a systematic review and meta-analysis

**DOI:** 10.1186/s13041-026-01323-0

**Published:** 2026-06-19

**Authors:** Fatemeh Sadat Gheibi, Leila Hosseini, Parinaz Kalejahi, Saeed Dastgiri, Ali Reza Shafiee-Kandjani, Seyed Gholamreza Noorazar

**Affiliations:** 1 Research Center of Psychiatry and Behavioral Sciences, Tabriz University of Medical Sciences, Tabriz, Iran; 2 Department of Community Medicine, Tabriz University of Medical Sciences, Tabriz, Iran

**Keywords:** AdipoRon, Cognition, Alzheimer, Depression, Anxiety, Meta-analysis

## Abstract

Rising cases of cognitive disorders, depression, and anxiety underscore the need for new treatments, given the limited effectiveness and side effects of current options. AdipoRon targets adiponectin receptors and shows promise for protecting the brain, reducing inflammation, and supporting metabolism. This review examines preclinical data to determine whether AdipoRon consistently improves mood and cognitive function and to identify the underlying neurobiological pathways. We conducted a comprehensive literature search using PubMed, Embase, Web of Science, and Scopus, with no time limit, up to August 30, 2025. The quality of the selected studies was evaluated using the Collaborative Approach to Meta-Analysis and Review of Animal Studies (CAMARADES) checklists and the SYRCLE risk of bias tool. The studies found that AdipoRon treatment significantly reduced immobility in the forced swim test and had a significant anxiolytic effect in the open field test, especially in chronic unpredictable mild stress models. It also improved recognition memory in the novel object recognition test in models of Alzheimer’s and Parkinson’s diseases. Additionally, AdipoRon increased the expression of synaptic proteins, such as synaptophysin and PSD-95, in rodent models of these diseases. It also modulated the production of inflammatory cytokines. This review establishes AdipoRon’s capacity to resolve depressive, anxious, and cognitive deficits in rodent models. Because the meta-analyses were based on a limited number of studies and substantial heterogeneity was observed across studies, the findings should be interpreted with caution. However, further well-designed preclinical and clinical investigations are essential to confirm these findings.

## Introduction

 Cognitive impairments, depression, and anxiety are three interlinked domains that can negatively affect an individual’s mental state and functional capacity in day-to-day activities [1]. Cognitive impairment is a common feature of neurological disorders, including dementia and traumatic brain injury, and of psychiatric disorders, including schizophrenia, depression, and anxiety [2]. In these patients, cognitive impairment varies across attention, processing speed, and difficulties with decision-making, problem-solving, or completing daily tasks. Some cognitive impairments are more visible in daily life and arise from complex interactions among discriminative and causal factors and independent neurocognitive models [3, 4].

Depression is characterized by symptoms such as a loss of interest in activities, feelings of sadness, and cognitive deficits, including difficulties with concentration and decision-making [1]. On the other hand, anxiety disorders often manifest as excessive and irrational worry, restlessness, psychomotor agitation, and increased physiological arousal, which can also lead to cognitive difficulties [2–4].

Adiponectin is a hormone produced by adipose tissue, and its relevance has become a major subject of medical interest owing to its diverse functions in human health [5]. This hormone has been linked to regulating various bodily processes, such as glucose regulation in the bloodstream, fatty acid metabolism, and other intricate cellular processes [5]. Adiponectin influences cellular functions through its interaction with two receptors: adiponectin receptor 1 (AdipoR1) and adiponectin receptor 2 (AdipoR2), which are central regulators of essential cellular function [6, 7]. These receptors are abundantly expressed in several brain regions, such as the hippocampus, cortex, brainstem, and hypothalamus [8]. Activation of AdipoR1 and AdipoR2 regulates several intracellular signaling cascades, primarily the AMP-activated protein kinase (AMPK)/Sirtuin 1 (SIRT1) and peroxisome proliferator-activated receptor-α (PPAR-α) pathways, which play critical roles in cellular energy homeostasis, mitochondrial function, inflammation inhibition, oxidative stress reduction, neuronal survival, and vasodilatory properties [9–11]. AdipoRon can traverse the blood-brain barrier and activate adiponectin-related pathways by interacting with both AdipoR1 and AdipoR2 [12]. As an orally active small-molecule AdipoR agonist, AdipoRon exerts various biological properties in peripheral and neural tissues, such as pro-neurogenic, insulin-sensitizing, anti-atherosclerotic, anti-depressant, anti-oxidant, and anti-inflammatory activities [12, 13]. Studies investigating the effects of AdipoRon on Alzheimer’s disease (AD) indicate that it exerts neuroprotective actions [13–15]. It could decrease neuroinflammation and tau hyperphosphorylation, and improve mitochondrial function [16, 17]. Moreover, research has shown that long-term administration of AdipoRon reverses cognitive deficits and attenuates pathological features in experimental models, including cell lines and transgenic mice [18]. It also promotes the expansion of hippocampal stem cell populations and enhances synaptic function through the AdipoR1/AMPK signaling pathway [19]. Overall, this body of evidence supports AdipoRon as a new treatment option that may address multiple harmful processes related to AD, thus improving cognition and slowing down progression [17]. Furthermore, studies have demonstrated that AdipoRon effectively alleviates anxiety and depression-like behaviors in animal models, highlighting its broader therapeutic potential in addressing psychiatric conditions [20].

A study examined the therapeutic effects of AdipoRon, a synthetic adiponectin receptor agonist, on anxiety and depression symptoms caused by chronic sleep restriction (SR) in mice. Chronic SR, defined as sleep of 6 h or less per night, is an increasing problem associated with mood disorders. The researchers used a mouse model subjected to 21 days of SR and provided daily intranasal treatment with AdipoRon, comparing it with melatonin and a control group. Behavioral tests (open field, elevated plus maze, forced swim, sucrose splash tests) showed that SR resulted in anxiety-like and depression-like behaviors. Intranasal AdipoRon notably decreased anxiety behaviors and depressive symptoms [21].

AdipoRon treatment effectively lowered corticosterone levels, suppressed microglial activation, and reduced inflammatory cytokine expression, indicating its anti-inflammatory properties are mediated by the suppression of the NF-κB signaling pathway. The study suggests that AdipoRon mediates its anxiolytic and antidepressant effects by regulating the hypothalamic-pituitary-adrenal (HPA) axis and neuroinflammation [22]. Based on the available evidence, AdipoRon appears to contribute to improved cognitive performance and to the reduction of depressive- and anxiety-like behaviors [18]. These findings suggest that AdipoRon may represent a promising candidate for future therapeutic strategies targeting cognitive disorders, depression, and anxiety. Accordingly, the present review examines and synthesizes the existing literature in this area.

## Method

### Search strategy

The search strategy carried out in this systematic review followed the Preferred Reporting Items for Systematic Reviews and Meta-Analyses (PRISMA) guidelines. The protocols used in this systematic review and meta-analysis have been registered in PROSPERO (CRD420251164652). The Ethics Committee of Tabriz University of Medical Sciences (IR.TBZMED.VCR. REC.1404.115) approved the research.

Two researchers independently searched PubMed, Embase, Web of Science, and Scopus for articles without a time limit until August 30, 2025. The relevant database, Open gray, will also be used to evaluate gray literature. The Search keywords based on MeSH terms were as follows:

(((((cognitive[Title/Abstract]) OR (cognitive impairment[Title/Abstract])) OR (“Cognitive Dysfunction“[Mesh])) OR (cognition[Title/Abstract])) OR (“Cognition“[Mesh]))) AND ((AdipoRon[Title/Abstract]) OR (Adiponectin receptor agonist[Title/Abstract]))) OR (((AdipoRon[Title/Abstract]) OR (Adiponectin receptor agonist[Title/Abstract])) AND (((Anxiety[Title/Abstract]) OR (“Anxiety“[Mesh])) OR (anxiolytic[Title/Abstract])))) OR (((AdipoRon[Title/Abstract]) OR (Adiponectin receptor agonist[Title/Abstract])) AND ((depression[Title/Abstract]) OR (“Depression“[Mesh]))). The PICO framework guiding our study was as follows:

(P)The population consists of preclinical models of depression, anxiety, and cognitive disorders, including animal studies.

(I)The intervention focuses on AdipoRon-based adiponectin replacement therapies for addressing depression, anxiety, or cognitive impairment.

(C) The comparison involves control groups, such as untreated models or those administered a placebo.

(O)The outcomes evaluated include depression-like or anxiety behaviors, neuroinflammation, cognitive function, molecular pathways, and the systemic therapeutic impact of AdipoRon.

### Inclusion criteria

In light of the lack of clinical trials, our study focused on preclinical research. We included studies employing animal models to investigate depression, anxiety, and cognitive impairments, in which experimental groups were treated with AdipoRon. Furthermore, inclusion was restricted to studies published in English to ensure consistency and accessibility of the data.

### Exclusion criteria

Exclusion criteria included: articles that did not meet the required quality, letters to the editor or proposals, case reports, studies, reprints that used the same sample data, conference abstracts, and narrative reviews.

### Data extraction

Data extraction was performed using a standardized template to gather key details: the first author, publication year, experimental models, behavioural tests, AdipoRon treatment specifics (including dosage and duration), and outcomes. Means, sample sizes, and standard deviation (SD) were obtained from studies. In some studies, multi-arm treatments were used, including different dosing regimens. We assumed each dose was an independent study. When data were only shown graphically, the GetData Graph Digitizer software (version 2.26) was used to extract numerical data. If SD was not reported, it was calculated as √N × SEM, where N is the sample size.

### Quality assessment

The CAMARADES (Collaborative Approach to Meta-Analysis and Review of Animal Data from Experimental Studies) quality checklist was employed in this systematic review. It provides a standardized framework for assessing methodological rigor and reproducibility. The checklist consists of ten essential items: (1) publication in a peer-reviewed journal, (2) statement regarding temperature control, (3) randomization of treatment or control, (4) allocation concealment, (5) blinded assessment of outcomes, (6) avoidance of anesthetics with significant intrinsic properties, (7) animal model description, (8) calculation of sample size, (9) compliance with animal welfare regulations, and (10) disclosure of potential conflicts of interest.

Also, we employed SYRCLE’s (Systematic Review Centre for Laboratory Animal Experimentation) tool to conduct a comprehensive bias risk assessment. This tool has 10 items covering common bias types. These include adaptations of five Cochrane domains (sequence generation, allocation concealment, incomplete data, selective reporting, other bias) plus five items tailored to animal studies (baseline characteristics, random housing, blinding of caregivers, random outcome selection, blinding of outcome assessment). Quality assessment was conducted independently by two authors (LH and FG), and differences were resolved through discussion by the third author (PK).

### Statistical analysis

The meta-analysis was conducted using RevMan version 5.4.1 (version 5.4; The Nordic Cochrane Centre, Copenhagen, Denmark). Effect sizes were reported as weighted mean differences (WMD) with 95% confidence intervals (CI) for comparisons between the treatment and control groups. Study heterogeneity was evaluated using the chi-square distribution and the *I²* statistic. A random effects model was implemented when *I*^2^ > 50%; otherwise, a fixed effects model was applied. Subgroup analyses were performed based on route of injection and disease. In all tests, *p*-values less than 0.05 were considered statistically significant.

## Results

### Study selection

**Fig. 1:**
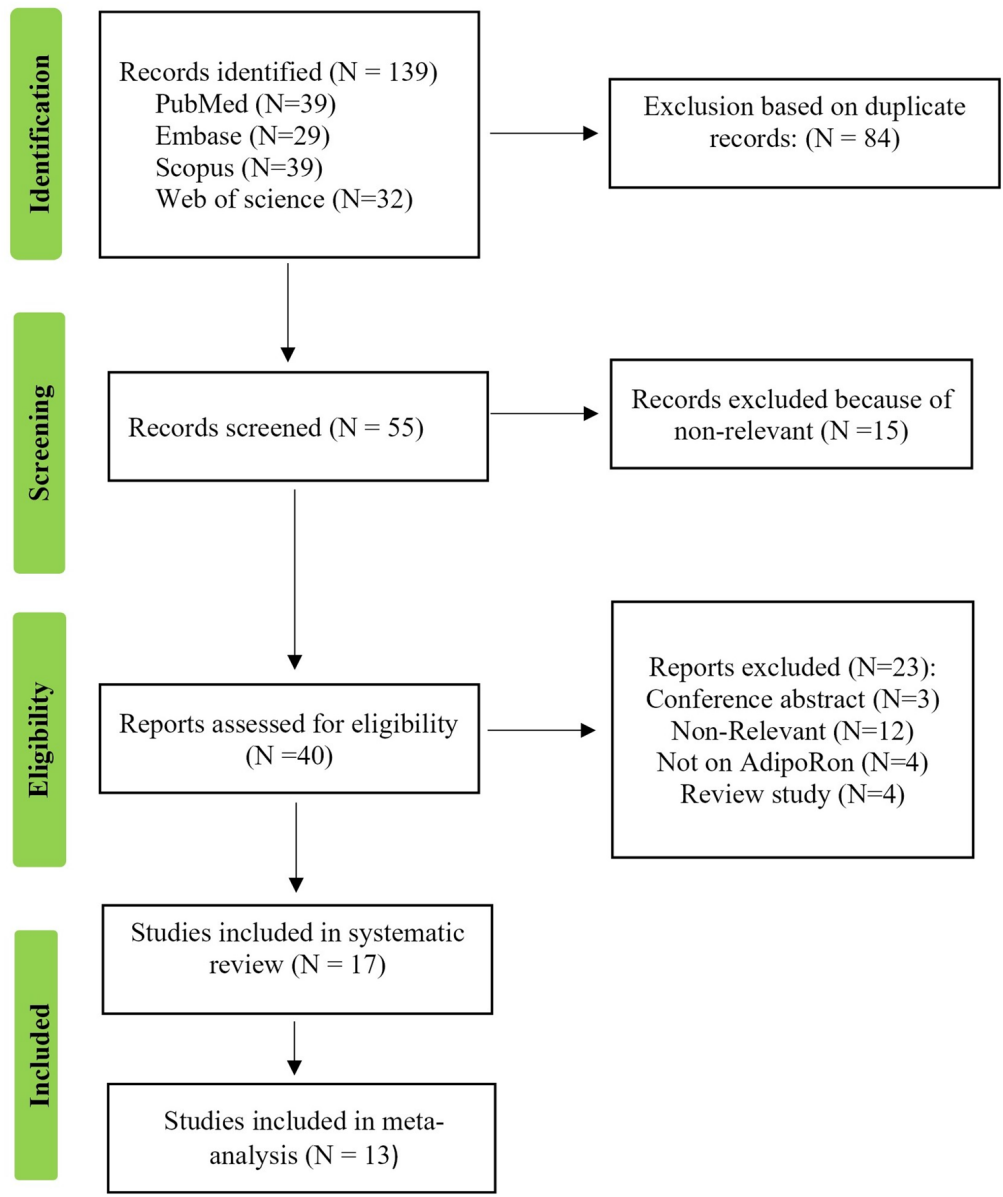
Flow diagram of the study identification and selection process according to PRISMA guidelines

A comprehensive literature search identified 139 articles. We excluded 84 articles that contained duplicate content. Fifty-five full-text articles were then assessed using inclusion and exclusion criteria. Of these, 17 studies met the inclusion criteria and were incorporated into this review. Figure 1 presents a PRISMA flow diagram outlining the study selection process.

### Characteristics of included studies

This systematic review and meta-analysis include a total of 17 studies published up to 2025, with their key characteristics summarized in Table 1. C57BL/6 mice were used in 10 studies [12, 18, 22–29], Wistar rats were used in 2 studies [20, 30], Swiss albino mice were used in only one study [21], P301S mice were used in only one study [31], APP/PS1 mice were used in 2 studies [13, 32], and 5xFAD mice were used in only one study [23]. The AdipoRon dosage ranged from 1 µg/kg to 50 mg/kg. Four studies administered AdipoRon via gavage [13, 23, 27, 31], seven via intraperitoneal injection [12, 18, 22, 23, 25, 26, 28], three via intranasal injection [20, 21, 30], and the remaining via intracerebroventricular (ICV) [24, 29, 32]. The duration of AdipoRon ranged from 7 days to 4 months. All studies used male animals.

**Table 1:** Characteristics of the included studies

Author and year	Type of study	Species	Sex age (months)	Duration, dose, and route of treatment	Anxiety	Depression	Cognitive	Main findings in ivivo
Douglas A. Formolo et al. 2023	Invivo	C57BL/6J and CamKIIα-Cre mice	Male, Eight-week-old	7-day, 20 mg/kg, i.p	Novelty-suppressed feeding test and Light–Dark Box	SPT and FST		Reduced immobility time in the FST, anxiolytic-like effects, no change in adult hippocampal neurogenesis, reduced protein levels of BDNF and PSD-95
Thomas H Lee et al. 2021	Invivo and invitro, T2DM	C57BL/6J Mice	Male, Five-week-old	20 mg/kg, i.p,14 days	OFT		Y-Maze test	Improved spatial memory, restored hippocampal adult neurogenesis, activated AMPK/PPAR-γ/PGC-1α signaling, ameliorated spine loss, restored LTP deficits in the dentate gyrus, promoted BDNF Levels, and increased serum adiponectin
Thomas H. Lee et al. 2021	In vivo, Memory	C57BL/6J Mice	Male, Five-week-old	20 or 50 mg/kg i.p., continuously for 14 days	OFT		Y-Maze test	20 mg/kg promoted hippocampal cell proliferation, increased serum levels of adiponectin and BDNF, no effects on spatial recognition memory and locomotor activity. At a dose of 50 mg/kg, impaired spatial recognition memory, inhibited cell proliferation, and neuronal differentiation
Bin Liu et al. 2020	In vivo, AD	C57BL/6 mice, APP/PS1	Male ,7 months	1 µg in 2 µl vehicle, lateral ventricle			OFT, NOR, Y-maze, and MWM	Suppressed the Aβ deposition and inhibited BACE1 expression in both the cortex and hippocampus, reversed the dissipation of the ΔΨm, escape latency decreased, and improved cognitive
Wenyan Zhao et al. 2025	In vivo, T2DM	C57BL/6 mice	Male, Seven-week-old	20 mg/kg and a vehicle i.p, 14 days	OFT	SPT and FST		Improved depression-like behavior, anti-apoptotic effect in the hippocampus, and increased the number of synapses in the prefrontal cortex and hippocampus, increased the density of dendritic spines in the CA1 region, decreased NLRP3, ASC, and IL-1β protein levels in the PFC and hippocampus, and increased the ratio of LC3II/ LC3I and beclin1 in the hippocampus, activated the AMPK/mTOR autophagy pathway, and increased AdipoR1
Mayuri Khandelwal et al. 2022	Invivo and invitro, AD	APP/PS1 mice	Male, Seven-week-old	50 mg/kg, gavage, 30 days			MWM and Y- maze	Potentiated insulin sensitivity, reversed cognitive deficits, reduced Aβ amyloid burden, and induced AMPK activation, reduced GFAB and promoted GLUTs expression, increased PSD-95 and synaptophysin, and BACE
Roy Chun-Laam Ng et al. 2021	Invivo and invitro, AD	5xFAD mice and wild-type C57BL/6 N	Male, Seven-week-old	50 mg/kg, gavage, 3 months	OFT		MWM, NOR , and the Fear-conditioning test	Improved cognitive functions and restored neuronal and synaptic densities, rescued neuronal loss, and restored reduction of dendritic spine density, decreased Aβ deposition, and ameliorated amyloid pathology, diminished microgliosis, astrogliosis, and inflammatory responses (IL1β and TNFα, GFAB)
Beriwan Malaei et al. 2025	In vivo, Sleep	Swiss albino mice	Male, 3 months old	10 µg/mouse in 10 µL, Intranasal, 21 days	OFT and EPM	FST and SPT		Attenuated depressive-like symptoms, decreased serum corticosterone, suppressed microgliosis in the PFC (Iba-1), inhibited inflammatory responses (IL-1β, p-NF-κB)
Sarah Nicolas et al. 2018	In vivo, Depression	C57BL/6J mice	Male, five-week-old	1 mg/kg, i.p, 3 weeks	NSF test	Light and dark test, SPT, social interaction test, learned helplessness test, FST		Ameliorated corticosterone, induced excess weight and dyslipidemia, reversed the anxiety and depressive-like behavior, restored BDNF, VEGFα, IGF1, and NGF
Fengjiao Sun et al. 2024	In vivo and in vitro, AD	APP/PS1 transgenic mice	Male, 8 months	1 µg per mouse, ICV, 7 days			NOR test, Y-maze, and MWM	Facilitated Aβ clearance, activated autophagy (LC3II, P62), AdipoR1/AMPK, and SIRT1, alleviated the cognitive impairment
Soraya Alimohammadi et al. 2025	Invivo, PD	Wistar Rat	Male, 2 months	0.1, 1, 10 µg in 10 µl/rat, Intranasal, 21 days			NOR and Barnes-Maze test	Improved recognition memory and motor function, spatial memory, decreased ROS levels, enhanced the antioxidant system (SOD, GPX, TAC), upregulated BDNF and PSD-95
Negin Azizifar et al. 2024	Invivo, PD	Wistar Rat	Male, 2 months	0.1, 1, and 10 µg, Intranasal, 21 days	OFT and EPM	SPT and FST		Reduced corticosterone, decreased inflammasome components (NLRP3, caspase 1, and IL-1β), and increased Sirt-1 protein levels in the prefrontal cortex
Yaqi Liu et al. 2024	In vitro and in vivo, Depression	C57BL/6J mice	Male,7–8 weeks old	10,20,40 mg/kg, i.p, 14 days	OFT	SPT, TST, and FST		Ameliorated depressive-like behaviors, alleviated neuronal damage and inflammation (IL-1β, IL-6, TNF-α, and IL-18), inhibited Iba-1, and NLRP3/caspase 1/IL-1β
Guangyang Bai et al. 2024	Invivo, Septic	C57BL/6J mice	Male, 8 weeks	50 mg/kg, gavage, 7 days			MWM	Improved memory deficits, ameliorated synapse damage and neuronal loss, promoted the phosphorylation of adenosine 5 ’-monophosphate activated protein kinase (pAMPK)
Wenyan Zhao et al. 2024	Invivo, T2DM, AD	C57BL/6 mice	Male, 8 weeks	50 mg/kg, i.p, 14 days			MWM	Restored the cognitive deficits, up-regulated PSD95, SYN, GAP43, and SYP, increased the number of hippocampal synapses, attenuated synaptic damage and Tau phosphorylation, decreased the activity of GSK-3β, increased AdipoR1&2, p-AMPK, AMPK, Reduced p-mTOR/mTOR
Cailin Wang et al. 2023	In vitro and in vivo, AD	SH-SY5Y cells, P301S mice	Male, 6 months old	20 µM in vitro, 50 mg/kg in vivo, gavage, 4 months			NOR and MWM	Improve synaptic function, enhanced mitochondrial fusion, and mitigated hyperphosphorylated tau accumulation, rescued memory deficits
Jingjing You et al. 2021	Depression	C57BL/6J mice	Male, 12-month- old	1 µl/1 mM, ICV	OFT, light-dark test, and EPM	FST, SPT, and TST	NOR, MWM, and Y-maze	Promoted hippocampal neurogenesis and improved cognitive dysfunction, increased the number of Ki67- and DCX-positive cells, increased expression of NICD, ADAM10, Hes1, Hes5, Hey1, and Heyl and upregulated Notch1 signaling

### Quality and risk of bias of included studies

**Fig. 2:**
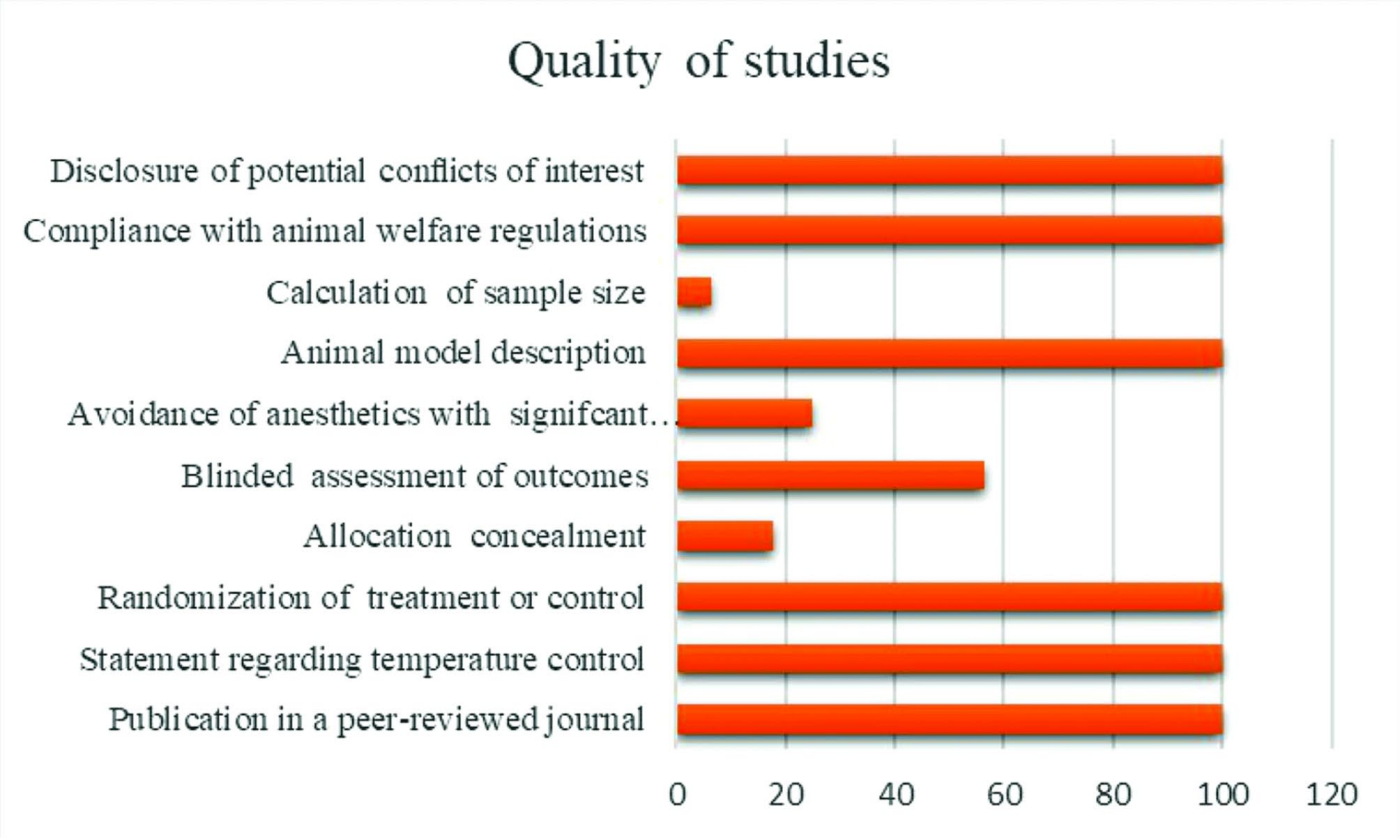
Evaluation of the included studies using the modified CAMARADES quality checklist

Of the studies reviewed, all were published in peer-reviewed journals and included statements on temperature control. A conflict of interest disclosure and mention of random allocation were provided in 100% of the articles. Three studies mentioned allocation concealment. All studies stated compliance with animal welfare regulations. Random allocation to treatment or control was reported in 100% of the publications. However, one article reported a sample size calculation. Blinded outcome assessment was described in 56.25% of the papers. Additionally, avoidance of anesthetics with significant intrinsic properties was reported in 25% of studies (Fig. 2).

### Risk of bias

**Fig. 3:**
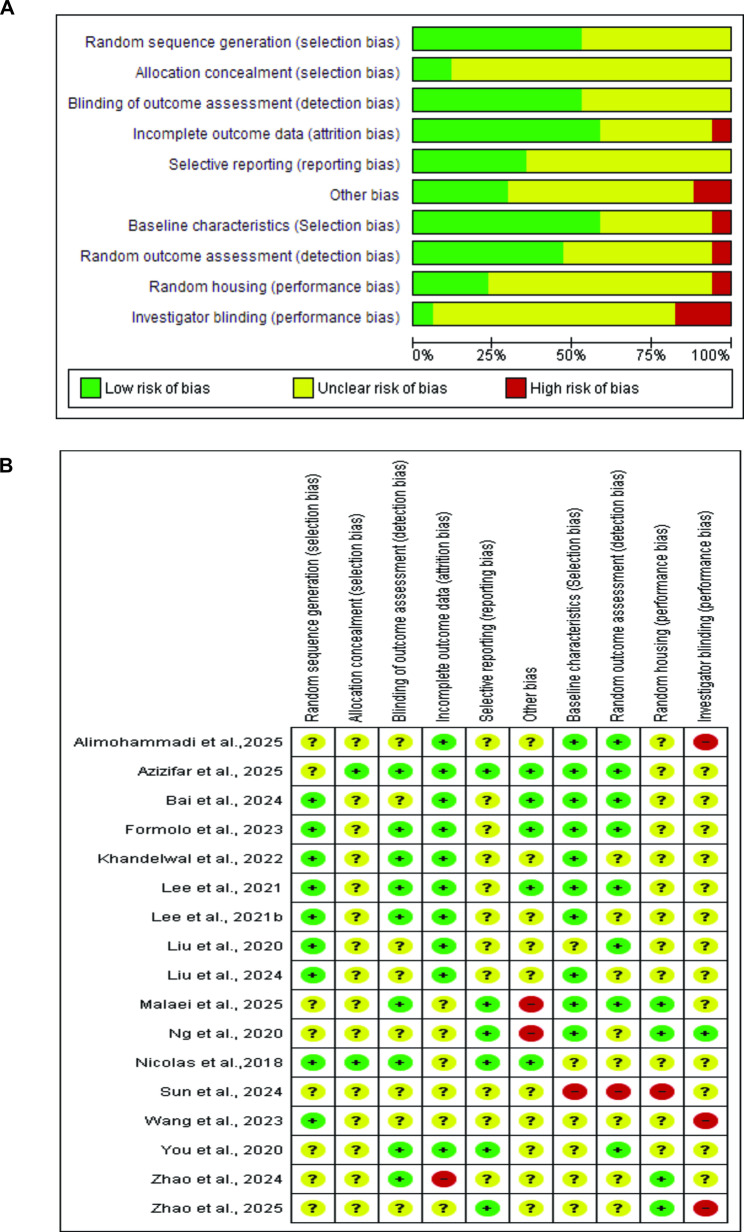
**A** Methodological quality graph: review authors’ judgments about each methodological quality item presented as percentages across all included studies. **B** Diagram of risk of bias in the included studies. + = low risk of bias, – = high risk of bias, ? = unclear risk of bias

The assessment of bias risk in the included studies indicated that the majority had either a low or moderate risk of bias across most domains. Sequence generation was deemed to be of low risk of bias in 52.94% of the studies. There was an unclear risk of bias regarding allocation concealment (88.23%), Selective outcome reporting (64.70%), other biases (58.82%), and Random housing (70.58%). High risk of bias was identified for investigator blinding in 17.65% of articles. Blinding of outcome assessment was low risk in 52.94% of the studies, and incomplete outcome data were low risk in 58.82% of the studies. Moreover, baseline characteristics (58.82%) and random outcome assessment (47.05%) were low risk in the studies (Fig. 3A and B).

### Behavioral tests

#### Depressive

##### Forced swim test (FST)

A total of three studies (7 arms) involving 66 animals in the AdipoRon group and 66 in the control group were included in FST [12, 20, 26]. The effects of AdipoRon were analyzed separately for Parkinson’s disease (PD) and depression models. In the subgroup analysis of PD models, three arms were included. AdipoRon administration resulted in a reduced immobility time versus controls (WMD = − 17.37, 95% CI [− 26.64,−8.10]; *p* = 0.0002). Heterogeneity among these studies was negligible (*I*^2^ = 0%), indicating a consistent effect across the included PD models.

**Fig. 4:**
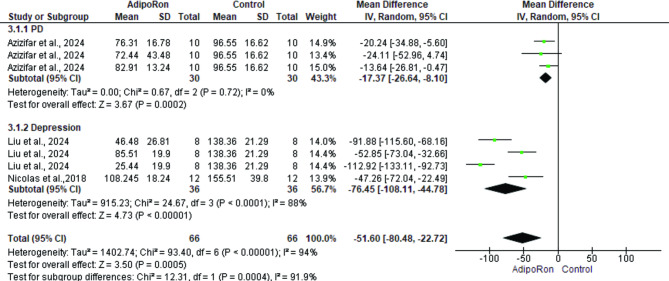
Forest plot of studies on the effect of AdipoRon on FST

Two studies (4 arms) were included in the depression model. AdipoRon significantly improved depressive-like behaviors (WMD = − 76.45, 95% CI [− 108.11, − 44.78]; *p* < 0.00001). Substantial heterogeneity was observed (*I²* = 88%), indicating marked variability across depression models. These findings are summarized in the forest plot shown in Fig. 4, which illustrates the effect of AdipoRon on FST.

##### Sucrose preference test (SPT)

**Fig. 5:**
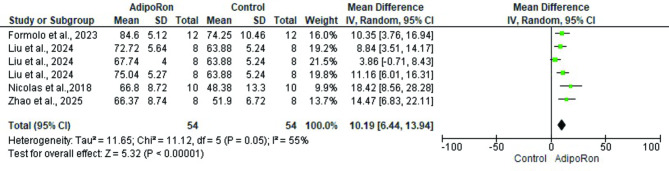
Forest plot of studies on the effect of AdipoRon on SPT

Regarding SPT, four studies (six arms) comprising 54 animals in the AdipoRon group and 54 animals in the control group were included in the meta-analysis of SPT [22, 25–27]. The results indicated that AdipoRon treatment significantly increased sucrose preference versus the control group (WMD = 10.19; 95% CI [6.44, 13.94]; *p* < 0.0001; *I*^2^ = 55%). Figure 5 presents the forest plot that demonstrates the effect of AdipoRon on SPT.

#### Anxiety

##### Open field test (OFT)

**Fig. 6:**
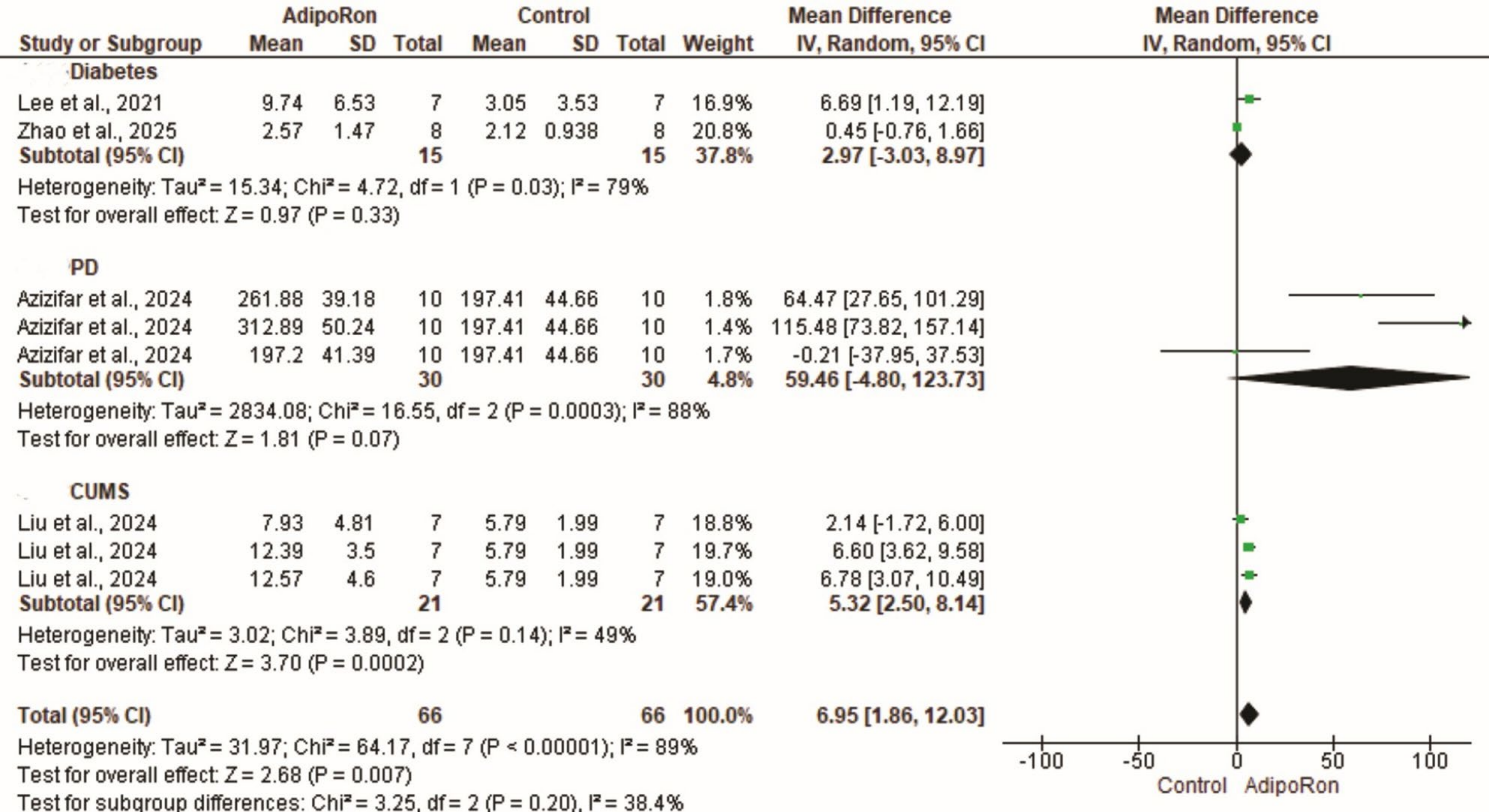
Forest plot of subgroup analyses for anxiety by spent center time in the OFT test

A random-effects meta-analysis was carried out to investigate the effect of AdipoRon on anxiety-like behavior in animal models, as measured by the OFT (Fig. 6). Eight arms of four studies from diabetes [23, 25], PD [20], and chronic unpredictable mild stress (CUMS) models [26] were included. In diabetic models, AdipoRon had a non-significant effect on anxiety-like behavior (WMD = 2.97, 95% CI [–3.03, 8.97], *p* = 0.33; *I*^2^ = 79%). In PD models, a non-significant trend was observed (WMD = 59.46, 95% CI [–4.80, 123.73], *p* = 0.07; *I²* = 88%). Conversely, in CUMS models, AdipoRon significantly reduced anxiety-like behavior (WMD = 5.32, 95% CI [2.50, 8.14], *p* = 0.0002; *I²* = 49%).

#### Cognition

##### Novel object recognition (NOR) test

**Fig. 7:**
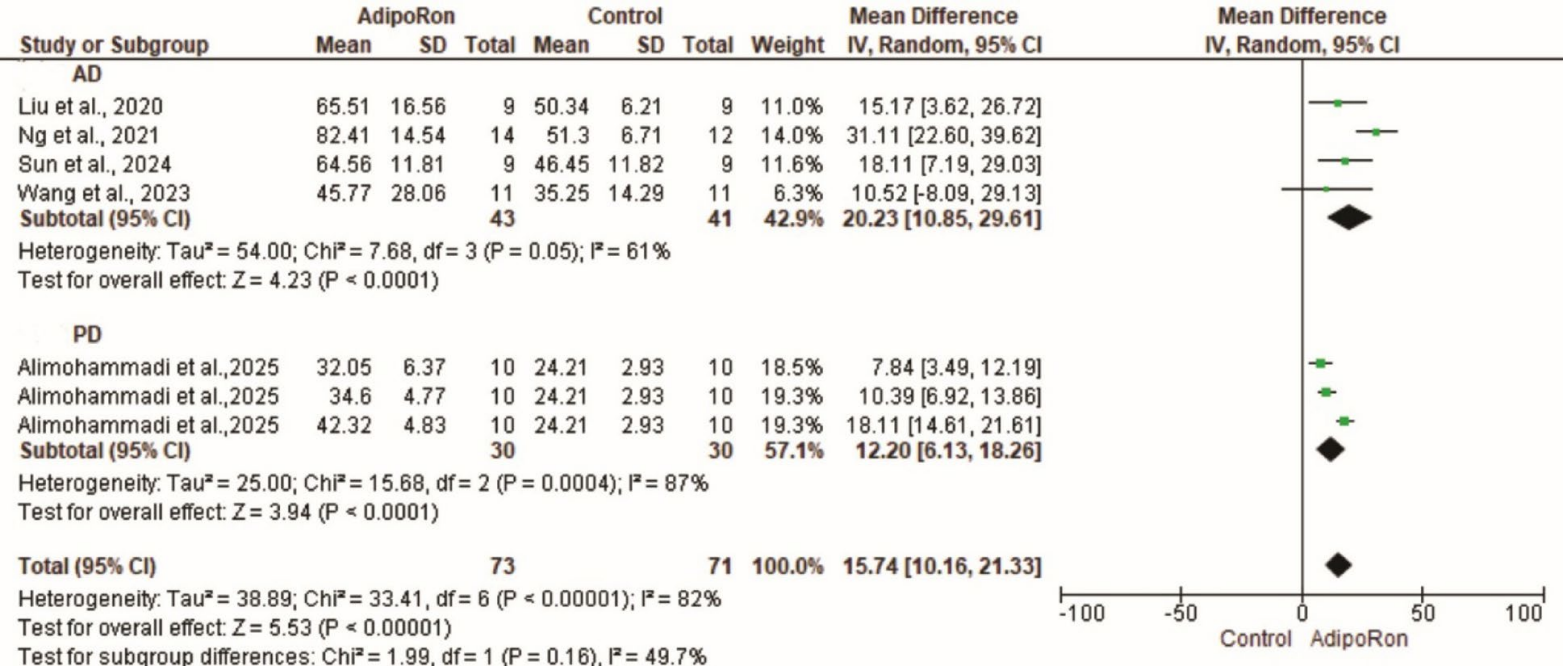
Forest plot of subgroup analyses for exploration time in the novel object in the NOR test

A random-effects meta-analysis was conducted to assess the impact of AdipoRon on recognition memory in animal models of neurodegenerative disorders (Fig. 7). In the AD model (4 studies) [8, 24, 31, 32], AdipoRon significantly improved NOR test performance compared with control groups (WMD = 20.23, 95% CI [10.85, 29.61], *p* < 0.0001; *I*^2^ = 61%). Similarly, in the PD subgroup, AdipoRon treatment could significantly improve (WMD = 20.12, 95% CI [16.13,18.26], *p* < 0.0001; *I*^2^ = 87%).

##### Effect of administration routes of AdipoRon on NOR performance

**Fig. 8:**
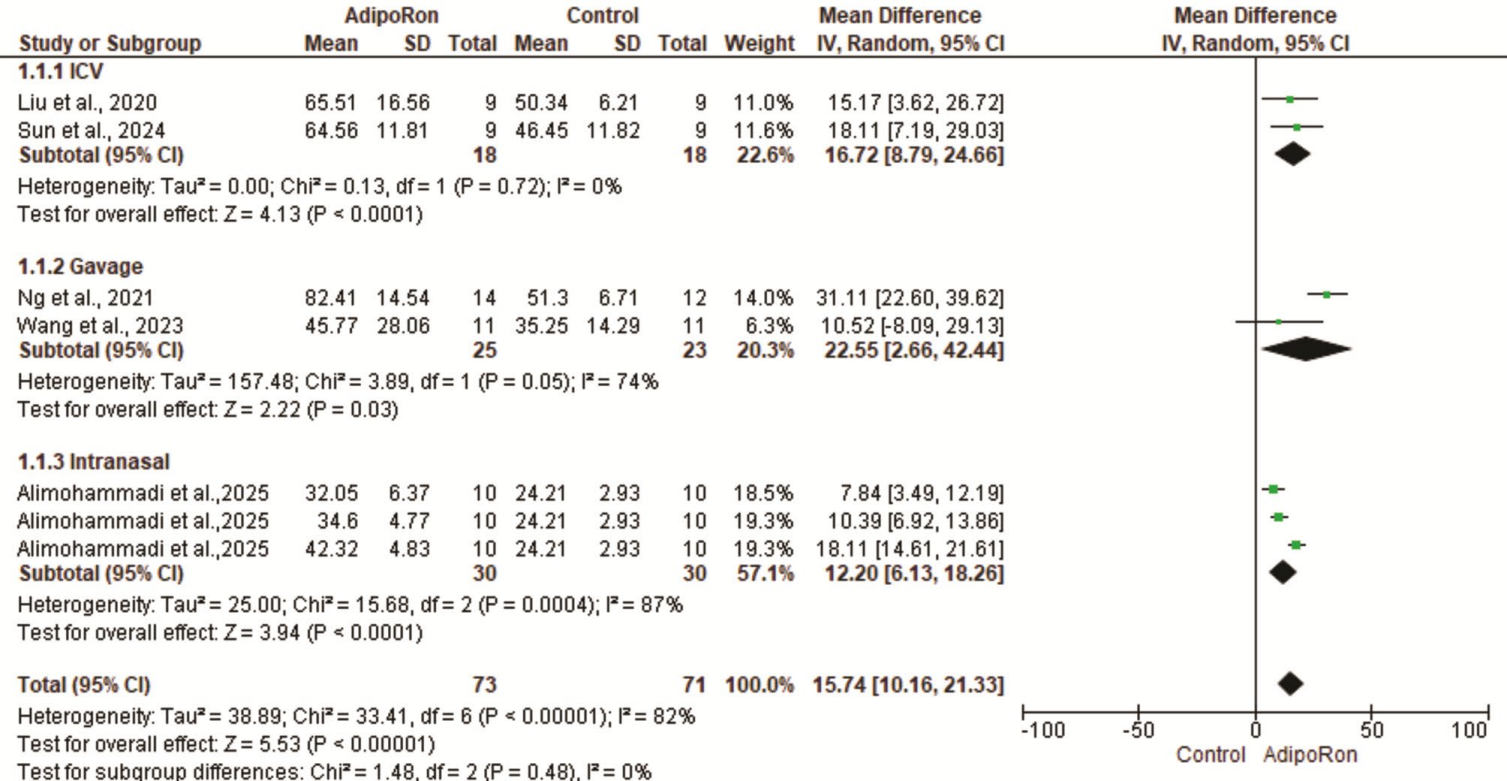
Forest plot of subgroup analyses for exploration time in the novel object in the NOR test. Grouped by route of AdipoRon injection

To investigate whether the route of administration influences the cognitive effects of AdipoRon, a subgroup meta-analysis was performed based on delivery methods (ICV, gavage, and intranasal). As seen in Fig. 8, for ICV injection, AdipoRon significantly increased exploration time for novel objects (WMD = 16.72, 95% CI [8.79,24.66], *p* < 0.0001; *I*^2^ = 0%).

Gavage AdipoRon administration also resulted in a significant positive effect (WMD = 22.55, 95% CI [2.66, 42.44], *p* = 0.03; *I*^2^ = 74%). Intranasal delivery of AdipoRon also showed improvement in recognition memory (WMD = 12.20, 95% CI [6.13, 18.26], *p* < 0.0001; *I*^2^ = 87%). Overall, AdipoRon treatment significantly improved recognition memory impairments compared with control groups.

##### Morris water maze (MWM) test

**Fig. 9:**
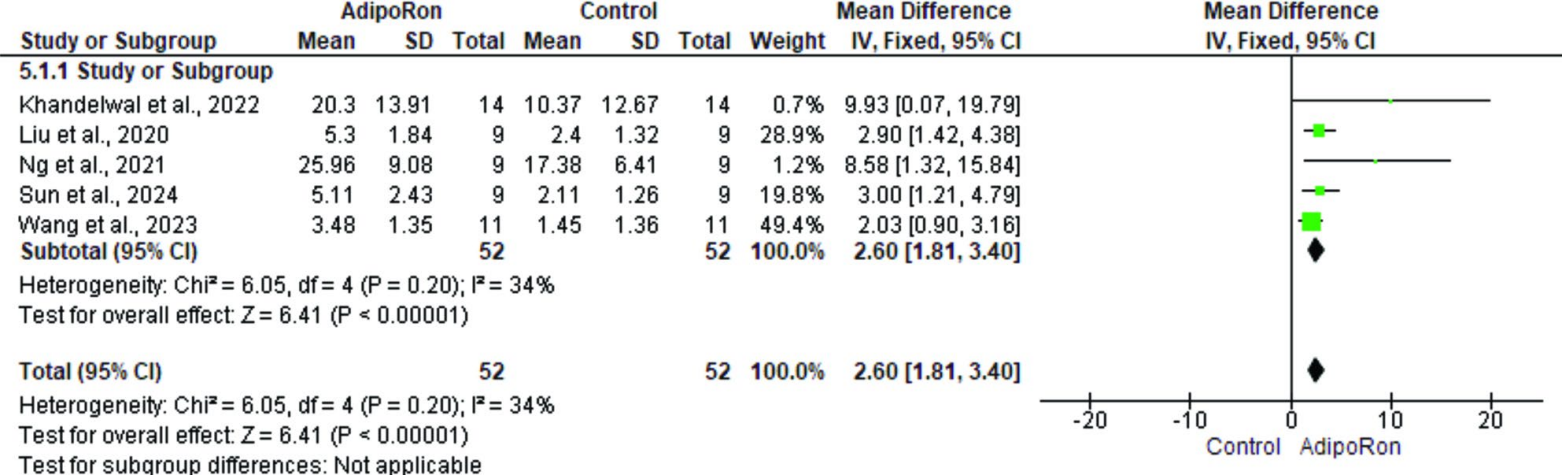
Forest plot of studies on the effect of AdipoRon on time spent in the target quadrant in the MWM test

Five studies were included in the meta-analysis of time spent in the target quadrant in the MWM test (*n* = 104: 52 treated animals and 52 control animals) [8, 13, 24, 31, 32]. Heterogeneity was moderate (*I*^2^ = 34%). AdipoRon-treated animals spent significantly more time in the target quadrant than controls (WMD = 2.60, 95% CI [1.81, 3.40], *p* < 0.00001). These results indicate that AdipoRon improves spatial learning in AD animal models (Fig. 9).

#### Neuroprotective mechanisms

#### Synaptophysin expression

**Fig. 10:**
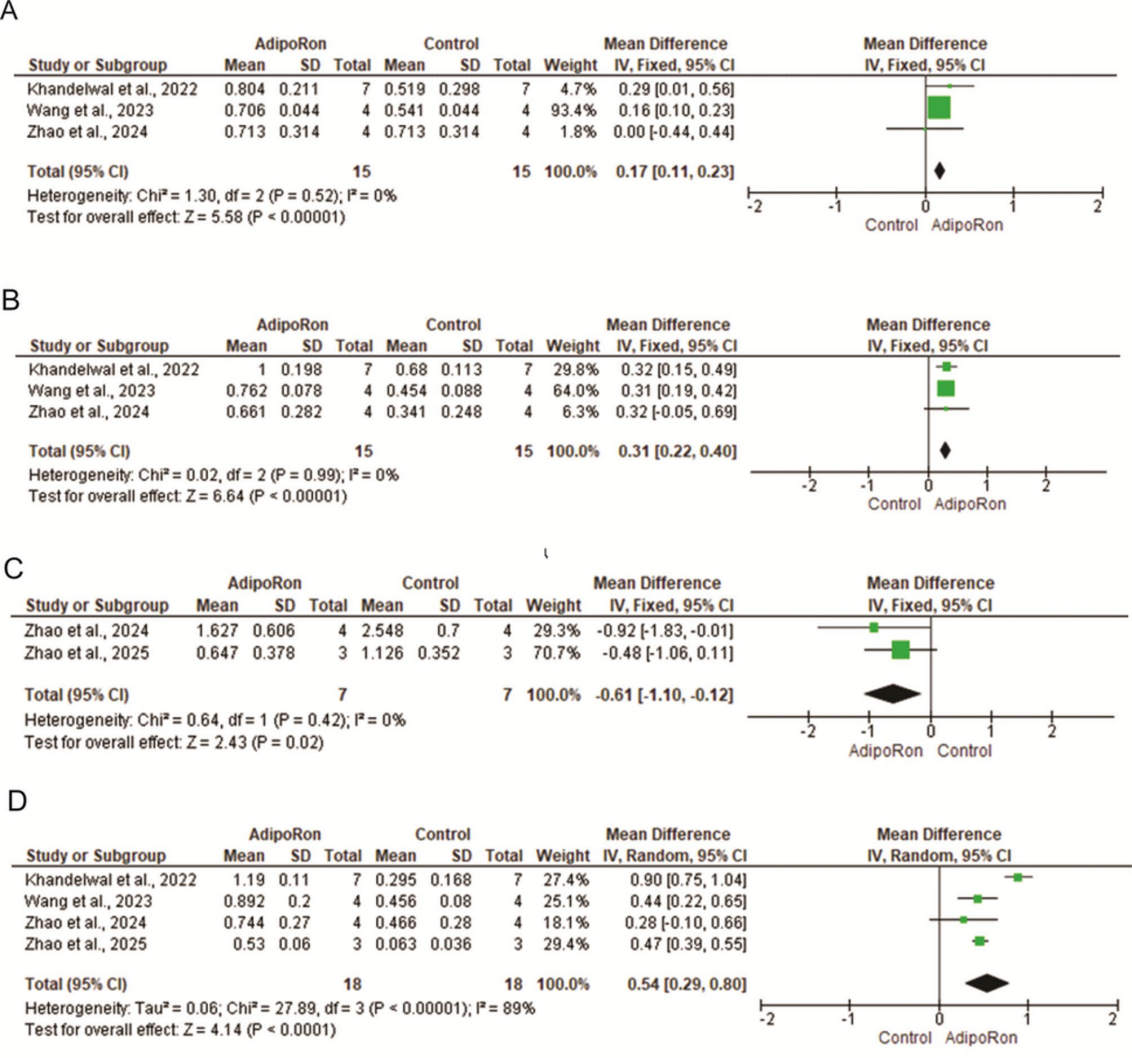
Forest plot of studies on the effect of AdipoRon on expression of **A** synaptophysin, **B** PSD-95, **C** P-mTOR /mTOR, and **D** P-AMPK/AMPK

For synaptophysin (SYP) expression levels, 3 studies were incorporated into the meta-analysis [13, 28, 31]. As seen in Fig. 10A, a significant increase in SYP expression levels was observed in animals treated with AdipoRon compared to the control group (15 treated animals, 15 control animals; WMD = 0.17, 95% CI [0.11, 0.23], *p* < 0.00001; *I*^2^ = 0%).

##### PSD-95 expression

We identified three studies that measured PSD-95 expression [13, 28, 31]. The pooled results showed a significant increase in PSD-95 levels in the treated group compared with the control group (15 treated animals and 15 control animals; WMD = 0.31, 95% CI [0.22, 0.40], *p* < 0.00001; *I*^2^ = 0%; Fig. 10B).

##### P-mTOR /mTOR expression

As seen in Fig. 10C, the analysis showed that AdipoRon treatment significantly reduced P-mTOR/mTOR expression versus the control group (7 treated animals, 7 control animals; WMD = − 0.61; 95% CI = [− 1.10, − 0.12]; *p* = 0.02; *I*^2^ = 0% ) [25, 28].

##### P-AMPK/AMPK expression

As shown in Fig. 10D, a meta-analysis of 4 studies [13, 25, 28, 31] showed that administration of AdipoRon could upregulate the expression of P-AMPK/AMPK ratio compared to the control group (18 treated animals, 18 control animals; WMD = 0.54; 95% CI = [0.29, 0.80]; *p* < 0.0001; *I*^2^ = 89%).

##### The effects of AdipoRon on memory in the Y-maze test

Of all included studies, six articles used the Y-maze test to evaluate hippocampal-dependent spatial recognition memory. Lee et al. [23] showed that administration of AdipoRon for 14 days restored the memory deficits in diabetic mice. In another study, treatment with 20 mg/kg of AdipoRon did not adversely affect spatial recognition memory; however, administration of 50 mg/kg resulted in a significant impairment of spatial memory, as evidenced by a marked reduction in the exploration index. These findings indicate that high-dose AdipoRon treatment may negatively influence spatial recognition memory [18]. According to Khandelwal et al. [13], AdipoRon treatment for 1 month could improve behavioral performance in the Y maze test by the percentage of time spent in the novel arm among AD mice. Moreover, APP/PS1 transgenic mice exhibited memory deficits that were ameliorated by intracerebroventricular injection of AdipoRon [24]. In the Y maze test, the APP/PS1 group demonstrated a reduced percentage of spontaneous alternation (SA%) compared to the wild-type group. Nevertheless, AdipoRon significantly enhanced the SA% in APP/PS1 transgenic mice [32].In a study, ICV injection of AdipoRon could not significantly affect SA% in depressed mice [29].

##### The effects of AdipoRon on anxiety in the elevated plus maze test (EPM)

Among the retrieved articles, two investigated the effect of AdipoRon on anxiety in the EPM test. A study by Malaei and colleagues revealed that the intranasal delivery of AdipoRon resulted in a significant both the time spent and entries into open arms compared with the control group in sleep-restricted mice [21]. Similarly, in PD animals, intranasal administration of AdipoRon (1 and 10 µg) increased open arm time and the number of open arm entrances in the EPM test [20]. Together, these findings suggest that intranasal AdipoRon effectively alleviates anxiety-related behavioral deficits.

##### The effects of AdipoRon on inflammation

Among the included studies, six articles evaluate the anti-inflammatory effect of AdipoRon. Nag et al. [8] provided evidence that AdipoRon reduced Iba-1 and activated astrocytes (GFAP) in both the cortex and hippocampus regions, and normalized the levels of pro-inflammatory factors, including interleukin (IL)-1β and tumor necrosis factor (TNF)-α, in AD mice. Similarly, Malaei et al. [21] found that treatment with AdipoRon attenuated the number of Iba-1-positive cells and decreased levels of TNF-α and IL-1β in the prefrontal cortex of sleep-restricted mice. Another study showed that AdipoRon reduced the density of GFAP-positive cells and Iba-1 1levels in the brain of transgenic APP/PS1 mice [13]. AdipoRon treatment was also shown to suppress the inflammasome pathway (NLRP3, caspase 1, and IL-1β) and reduce the levels of IL-6, TNF-α, and IL-18 in the hippocampus [12, 20, 26].

##### Effects of AdipoRon on corticosterone levels

Three studies also investigated the effects of AdipoRon on the corticosterone levels. The study by Nicolas et al. revealed that a three-week administration of AdipoRon reduced corticosterone levels in depressed mice [12]. Consistent findings have also been obtained from other studies [20, 21].

## Discussion

The current systematic review and meta-analysis support the beneficial effects of AdipoRon on behavioral and neuroprotective outcomes in rodent models. Based on the included studies, these improvements appear to be mediated primarily by upregulation of synaptic proteins, reduction of oxidative stress, and modulation of neuroinflammation. This study synthesized findings from preclinical research examining the antidepressant- and anxiolytic-like effects of AdipoRon, as well as its impact on cognitive function, across a range of behavioral and biochemical measures. The findings demonstrated that AdipoRon had a significant impact on behavioral domains, including immobility time in the FST, time spent in the central area in the OFT, and sucrose preference in the SPT, suggesting its efficacy in relieving depressive and anxiety symptoms in animal studies. Moreover, the administration of AdipoRon improves cognitive impairments in CNS diseases, such as AD, PD, and depression, as assessed by the MWM, NOR, and Y-maze tests.

### AdipoRon: depression and anxiety

The majority of the included studies that conducted depression assessment tests discovered that AdipoRon alleviates depression-like behaviors in PD and depression models in both mice and rats [12, 20, 22, 25, 26]. The pooled results of the current meta-analysis were consistent with most of these studies’ findings. A meta-analysis of FST results from studies revealed that AdipoRon reduced immobility time across experimental models [12, 20, 26]. In the PD model, AdipoRon could significantly decrease immobility time in the FST, with negligible heterogeneity (*I*^2^ = 0%). In contrast, the depression subgroup showed considerable heterogeneity (*I*^2^ = 88%), indicating substantial variability across studies. Several factors may explain this heterogeneity. First, different depression induction paradigms, including chronic unpredictable stress and corticosterone exposure, likely engage distinct neurobiological pathways. Second, variations in AdipoRon dosage and treatment duration may contribute to inconsistent outcomes. Despite this variability, the direction of effect remained consistently favorable, reinforcing the potential antidepressant properties of AdipoRon. Moreover, the findings of the SPT further support the antidepressant-like effects of AdipoRon in preclinical models. AdipoRon treatment significantly increased sucrose preference compared with controls, indicating attenuation of anhedonia, which is considered one of the core behavioral manifestations of depression [33].

D. A. Formolo et al. have demonstrated antidepressant- and anxiolytic-like effects after just 7 days of subchronic AdipoRon treatment, independent of hippocampal structural changes and synaptic function [22]. As Nicolas reported, mice under long-term corticosterone treatment showed increased immobility in the FST, reflecting reduced depressive-like states in mice [12]. The antidepressant effects of AdipoRon appear to be mediated through the modulation of neuroinflammation, as evidenced by alterations in IL-1β, IL-6, and TNF-α levels, as well as the normalization of indoleamine 2,3-dioxygenase and kynurenine aminotransferase expression. Furthermore, AdipoRon restored levels of brain-derived neurotrophic factor (BDNF), vascular endothelial growth factor-α, insulin-like growth factor-1, and nerve growth factor, while also increasing serotonin release and turnover [12].

Neuroinflammation is a key factor in the pathophysiology of depression. Specifically, inflammation mediated by microglia activation is believed to be closely associated with the onset and progression of depressive disorders [34]. Microglia-dependent activation of the inflammasome is crucial in the brain, and the NLRP3 inflammasome is identified as a pivotal molecular platform that regulates the release of pro-inflammatory cytokines. Activation of NLRP3 by repeated stress recruits ASC, which in turn activates caspase-1, which in turn promotes the cleavage of pro-IL-1β and pro-IL-18 into their mature forms [35]. Consequently, this process triggers an inflammatory response that can harm neurons and worsen disease progression [36]. Moreover, increasing evidence suggests that the NLRP3 inflammasome is hyperactivated in both patients with depression and in animal models of the condition [37, 38]. It has been reported that AdipoRon attenuated anxiety- and depression-like related behaviors in the PD rat model through inhibition of NLRP3 activation and modulating downstream inflammatory cascades [20]. You and colleagues examined the impact of intraventricular cannulation and ICV administration of AdipoRon in mice with depression. The findings indicated that AdipoRon enhances hippocampal neurogenesis and ameliorates depression-related cognitive deficits, primarily through the upregulation of Notch signaling and the expression of key regulatory genes [29].

Malaei et al. [21] found that intranasal AdipoRon treatment increased time spent in the open arms during the EPM test, diminished immobility time, and improved grooming behavior during the FST in mice exposed to chronic sleep deprivation, reducing anxiety and depressive symptoms.

A recent study by Li et al. [39] demonstrated that AdipoRon ameliorates anxiety- and depression-like behaviors in chronically stressed mice through modulation of the AMPK-PPAR2-BDNF-TrkB signaling pathway in the hippocampus. Although this study was not included in our meta-analysis due to its recent publication, it provides converging support for a critical role of the AdipoR2 signaling pathway in stress-related depression models. This mechanistic understanding of reported associations enhances the biological viability and translational significance of the outcomes from our review.

### AdipoRon and cognitive

The meta-analysis revealed that treatment with AdipoRon improves cognitive and behavioral performance. This is evidenced by increased time spent in the target quadrant in the MWM test and enhanced recognition memory in the NOR test. Accumulating evidence from in vivo models of AD, PD, and diabetes has investigated the efficacy of AdipoRon on cognitive function. They found that AdipoRon exerts beneficial effects on cognitive function via diverse molecular pathways. A study by Wang et al. [31] reported that AdipoRon treatment in SH-SY5Y cells and P301S transgenic male mice attenuated tau hyperphosphorylation and improved cognitive performance, synaptic function, and mitochondrial fusion, primarily through activation of the AMPK/GSK3β signaling cascade and upregulation of mitochondrial fusion proteins. A key limitation of the study is the four-month duration of AdipoRon treatment in mice, which may not be readily applicable to human clinical protocols and raises concerns regarding clinical feasibility and long-term safety.

The findings of Liu et al. [26] indicate that AdipoRon improves cognitive performance and reduces amyloid-β (Aβ) deposition in NE-4 C cells and APP/PS1 mice via the AMPK/CREB signaling pathway. Clinically, these results suggest that AdipoRon may promote neuronal survival and improve cognitive performance in AD by supporting neuroplasticity and mitigating amyloid-induced toxicity. Limitations on the clinical translatability of these findings include limited in vivo investigation time (7 days) and the methodology of administration via lateral ventricle injection, which may not be representative of longer-term human therapeutic dosing. Future studies should evaluate longer treatment durations and develop more suitable delivery strategies. Ng and colleagues [8] evaluated the effects of AdipoRonin in 5xFAD transgenic mice. They found that AdipoRon improved spatial memory and synaptic function and attenuated inflammation. These neuroprotective effects were mechanistically mediated through AMPK activation and the inhibition of Beta-Secretase 1 and NF-κB signaling.

The hippocampus plays a crucial role in regulating learning and memory processes, synaptic plasticity, and neuronal survival, mainly through high levels of BDNF expression. BDNF provides protection against neurotoxic damage, promotes the secretion and recycling of presynaptic dopamine, and addresses mitochondrial dysfunction in dopaminergic neurons of the substantia nigra [40]. Conversely, in individuals with PD and in animal models, there is a notable down-regulation of both the BDNF gene and its protein in the brain and blood. This reduction results in insufficient neurotrophic support, contributing to motor impairments as well as cognitive and psychiatric disorders [41, 42]. Research indicates that the accumulation of α-synuclein in the hippocampus reduces BDNF expression and synaptic proteins, including PSD-95(postsynaptic marker). This decline contributes to synaptic dysfunction and subsequent memory impairment. Alimohammadi et al. reported that adiporon at a dose of 10 mg/kg increases BDNF and PSD-95 levels [30]. Age-related decreases in the levels of PSD-95 are indicative of neuronal and synaptic loss in AD, which is associated with elevated amyloid burden and tau hyperphosphorylation. Additionally, a reduction in the SYP ( presynaptic marker) further exacerbates memory deficits in AD [43]. Several studies have demonstrated that AdipoRon administration increases PSD-95 and SYP levels in AD animals [13, 28, 31]. Moreover, AdipoRon treatment in diabetic mice restored hippocampal neurogenesis, increased spine density, facilitated hippocampal long-term potentiation, and increased serum and BDNF levels in the hippocampus [23].

A study by Lee et al. [18] demonstrated that while low-dose AdipoRon (20 mg/kg) enhanced hippocampal neurogenesis and spatial memory, a higher dose (50 mg/kg) unexpectedly impaired spatial memory, suggesting dose-dependent biphasic effects on hippocampal signaling. The high dose reduced serum adiponectin levels and BDNF, and the number of Ki67-, DCX-, and BrdU-positive cells, indicating impaired neurogenesis. Because BDNF plays a critical role in neuronal survival, synaptic plasticity, and memory formation, its reduction may contribute to the observed cognitive deficits [44]. One possible mechanism involves overactivation of the AdipoR1/AMPK signaling pathway at high doses of AdipoRon. Although AMPK activation is generally considered neuroprotective, AMPK hyperactivation may suppress mTOR signaling, reduce BDNF levels, and so impair hippocampal neurogenesis under physiological conditions. Furthermore, elevated corticosterone levels induced by high-dose AdipoRon may further contribute to reduced neurogenesis and memory impairment [45].

Recent evidence supports the beneficial effects of adiponectin signaling on cognitive function. Hui GG et al. [46] showed that three weeks of physical exercise (voluntary wheel running) increases adiponectin to increase Protein phosphatase 2 A (PP2A) activity, leading to reduced Tau protein phosphorylation levels in the hippocampus and improved cognitive deficits and neuroplasticity in chronic stress exposure mice.

AdipoRon has potential therapeutic implications for the treatment of AD, PD, and depression based on current scientific evidence; however, current limitations in our knowledge of the interactions between AdipoRon and various pathophysiological mechanisms of these diseases could prevent accurate translation from animal models to humans. Also, very few studies have employed long-term administration of AdipoRon, which hampers our ability to assess its long-term effects as a therapeutic agent for these diseases, given the need for consistent therapeutic intervention in chronic, progressive diseases. Future research should focus on conducting prolonged, longitudinal in vivo experiments using various model systems and populations to determine the safety, efficacy, and durability of AdipoRon as a therapeutic agent in humans.

In addition, this review is limited by the predominance of studies conducted in male rodents, which may constrain the external validity of the findings. Given the increased prevalence of depression in females and the potential for sex-specific mechanisms that contribute to depression in females, this imbalance reduces the translational applicability of the evidence. Future preclinical research should examine both sexes and systematically evaluate sex as a biological variable to enhance the robustness and clinical relevance of the findings.

## Conclusion

In this preclinical systematic review, AdipoRon demonstrated beneficial effects on depressive- and anxiety-like behaviors and improved cognitive performance in rodent model studies. These effects appear to be mediated through multiple mechanisms, including anti-inflammatory actions, modulation of neurotransmitter systems, and improvement of mitochondrial function. However, due to the lack of human studies available, the clinical applicability of adipoRon remains uncertain. Future research is needed to evaluate these findings through translational research designs and to develop agonists of adiponectin receptors with more favourable pharmacokinetic and safety profiles for future use as clinical therapeutic agents.

## Data Availability

No datasets were generated or analysed during the current study.

## Abbreviations

AdipoR1: Adiponectin receptor 1
AdipoR2: Adiponectin receptor 2
AD: Alzheimer's disease
BDNF: Brain-derived neurotrophic factor
CAMARADES: Collaborative approach to meta-analysis and review of animal studies
CI: Confidence intervals
CUMS: Chronic unpredictable mild stress
EPM: Elevated plus maze test
FST: Forced swim test
HPA: Hypothalamic-pituitary-adrenal
MWM: Morris water maze
NOR: Novel object recognition
OFT: Open field test
PD: Parkinson’s disease
PRISMA: Preferred reporting items for systematic reviews and meta-analyses
SPT: Sucrose preference test
SR: Sleep restriction
SYP: Synaptophysin
TNF: Tumor necrosis factor
WMD: Weighted mean differences
